# Co-receptors are dispensable for tethering receptor-mediated phagocytosis of apoptotic cells

**DOI:** 10.1038/cddis.2015.140

**Published:** 2015-05-28

**Authors:** B Park, J Lee, H Moon, G Lee, D-H Lee, J Hoon Cho, D Park

**Affiliations:** 1 School of Life Sciences and Bio Imaging Research Center, Gwangju Institute of Science and Technology, Gwangju 500-712, Korea; 2 Research Center for Cellular Homeostasis, Ewha Womans University, Seoul 120-750, Korea; 3 Department of Surgery and Pharmacology and Cell Biology, School of Medicine, University of Pittsburgh, Pittsburgh, PA 15213, USA; 4 Department of Biology Education, College of Education, Chosun University, Gwangju 501-759, Korea

## Abstract

During efferocytosis, phagocytic cells recognize dying cells by receptors binding to ligands specifically exposed on apoptotic cells. Multiple phagocytic receptors and some of their signaling pathways have been identified. However, the downstream pathways of tethering receptors that secure apoptotic cells remain elusive. It is generally assumed that tethering receptors induce signaling to mediate engulfment via interacting with co-receptors or other engulfment receptors located nearby. However, it is poorly understood whether co-receptors for tethering receptors exist during efferocytosis, and, if they do, whether they are indispensable for this process. Here, we address this issue using glycophosphatidylinositol (GPI)-anchored annexin A5 (Anxa5-GPI), an artificial tethering receptor without a putative co-receptor. Phagocytes expressing Anxa5-GPI exhibited enhanced binding of apoptotic cells, resulting in promoted ingestion of apoptotic cells in a phosphatidylserine-dependent manner. Anxa5-GPI-induced phagocytosis of apoptotic cells relied on the known cytoskeletal engulfment machinery but partially depended on the Elmo-Dock-Rac module or the integrin pathway. In addition, Anxa5-GPI-mediated efferocytosis provoked anti-inflammatory responses. Taken together, our work suggests that co-receptors are dispensable for tethering receptor-induced efferocytosis and that tethering receptors mediate the engulfment of apoptotic cells through multiple engulfment signaling pathways.

The removal of apoptotic cells, known as efferocytosis, is a series of arranged events from the recruitment of phagocytes to sites where apoptotic cells are generated to the digestion of apoptotic cells by phagocytes.^1, 2, 3^ One of the key steps during efferocytosis is the recognition of dying cells by phagocytes. Phagocytes can detect apoptotic cells by the direct or indirect association of multiple receptors on phagocytes with ligands on apoptotic cells.^4, 5, 6, 7, 8, 9^ Some receptors on the surface of phagocytic cells not only bind to apoptotic cells but also transduce apoptotic cell recognition signals into phagocytes in order to mediate the ingestion of apoptotic cells. For instance, brain-specific angiogenesis inhibitor 1 (BAI1) and stabilin-2, which are phosphatidylserine (PtdSer) receptors, recognize PtdSer on apoptotic cells and relay signals to the Elmo-Dock-Rac module and Gulp, respectively, via their cytoplasmic tails.^8, 10, 11^ By contrast, it has been suggested that other receptors, called tethering receptors, merely tether apoptotic cells to phagocytes without mediating downstream signal transduction, following which the internalization of apoptotic cells is mediated by the association of these receptors with co-receptors or other engulfment receptors located nearby.^12, 13, 14, 15, 16^ However, it is unclear whether co-receptors for tethering receptors exist in tethering receptor-mediated phagocytosis of apoptotic cells, and, if they do, whether they are indispensable for this process.

One intriguing characteristic of tethering receptors is that they have cytoplasmic tails lacking any signaling motifs or are anchored via glycophosphatidylinositol (GPI) to the outer leaflet of the plasma membrane. For example, Tim-4, a PtdSer receptor with a short cytoplasmic tail that promotes the engulfment of apoptotic cells by the binding of its IgV domain to PtdSer on apoptotic cells, lacks signaling motifs in its cytoplasmic tail. It has been known that neither the cytoplasmic tail nor the transmembrane region of Tim-4 is essential for Tim-4-mediated engulfment of apoptotic cells. Accordingly, it functions as a tethering receptor to secure apoptotic cells on phagocytes.^9, 14^ CD14 is located at the exofacial leaflet of the plasma membrane through its GPI anchor, which rules out the possibility that it mediates direct signal transduction into phagocytes after binding to apoptotic cells. Consequently, it is also considered to be a tethering receptor.^15^

Phospholipids such as PtdSer and phosphatidylcholine (PtdCho) are unequally distributed between the inner and outer leaflets of the plasma membrane in the normal state. For instance, uncharged phospholipids such as PtdCho and sphingomyelin are primarily located in the outer leaflet, whereas positively or negatively charged phospholipids (such as phosphatidylethanolamine or PtdSer, respectively) are restricted to the inner leaflet facing the cytosol.^17, 18, 19^ However, this asymmetric distribution of phospholipids in the plasma membrane is disrupted during apoptosis. In the plasma membrane of apoptotic cells, PtdSer is exposed to the outer leaflet of the plasma membrane by the activity of scramblases and flippases.^18, 20, 21^ Thus, exposed PtdSer is a hallmark of apoptotic cells and is the best characterized ligand on apoptotic cells for efferocytosis. PtdSer on the surface of apoptotic cells can be recognized by various PtdSer-sensing membrane proteins on phagocytes, collectively called PtdSer receptors, including tethering receptors.

Besides PtdSer receptors, many PtdSer-binding proteins have been identified. These proteins are involved in various biological processes such as blood coagulation, synaptic vesicle fusion, membrane scaffolding, and signal transduction.^22^ One of the best known proteins is annexin A5, which has been extensively studied as a PtdSer-binding protein. Annexin A5 belongs to the family of annexins, which are characterized by their Ca^2+^-dependent ability to bind to negatively charged phospholipids and share structural properties. Annexins are considered to be cytosolic proteins because they lack a 5′ leader sequence; however, some annexins, including annexin A5, are also found on the cell surface and in the circulation. This and related properties imply that annexins participate in diverse biological events from membrane dynamics to cell differentiation and migration.^23, 24, 25^ However, the physiological significance of this family is poorly understood. Among annexins, annexin A5 binds to PtdSer with high affinity. Because of this property, annexin A5 has been harnessed as a molecular probe to distinguish apoptotic cells from live cells both *in vivo* and *in vitro* for decades.^25, 26^

In this study, annexin A5 was expressed on the cell surface through a GPI anchor to delineate whether a tethering receptor without its co-receptor can promote efferocytosis. GPI-anchored annexin A5 (Anxa5-GPI) should not interact with any plasma membrane or extracellular protein, at least those involved in the engulfment of apoptotic cells. Thus, it is possible to exclude the effects of co-receptors on Anxa5-GPI-mediated phagocytosis of apoptotic cells. The expression of Anxa5-GPI in phagocytes promoted not only the binding but also the internalization of apoptotic cells. By contrast, phagocytosis of carboxylate beads and *Escherichia coli* was not affected by the expression of Anxa5-GPI in phagocytes. Anxa5-GPI-induced efferocytosis was not only partially dependent on a specific engulfment pathway but also relied on the generally known cytoskeletal engulfment machinery. Our observations suggest that co-receptors are dispensable for tethering receptor-mediated efferocytosis. In addition, tethering receptors could enhance efferocytosis through diverse engulfment machinery located nearby.

## Results

### Generation of Anxa5-GPI and its expression on the cell surface

**Figure 1:**
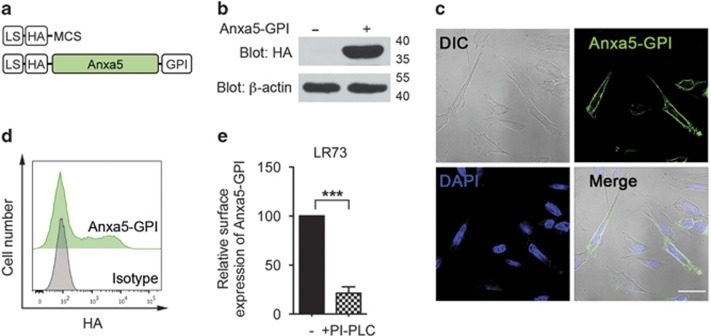
Annexin A5 fused to the GPI anchor signal of DAF is expressed in the exofacial leaflet of the plasma membrane. (**a**) Schematic diagram of the annexin A5 chimera protein. LS, leader sequence; HA, hemagglutinin A epitope; MCS, multi-cloning site; Anxa5, annexin A5; GPI, the GPI anchor signal of DAF. (**b**) LR73 cells were transfected with Anxa5-GPI and expression of Anxa5-GPI was detected with anti-HA antibody. *β*-actin was used as a loading control. (**c** and **d**) LR73 cells transfected with Anxa5-GPI were stained with anti-HA antibody and subsequently anti-mouse secondary antibody conjugated with Alexa 488 fluorochrome. Expression of Anxa5-GPI on the cell surface was evaluated using confocal microscopy (**c**) and flow cytometry (**d**). Scale bar, 20 *μ*m (**c**). (**e**) LR73 cells transfected with Anxa5-GPI were treated with PI-PLC at 4 ^o^C for 20 min. After that, the cells were stained for Anxa5-GPI without permeabilization and the expression of Anxa5-GPI on the cell surface was evaluated by flow cytometry. Mean fluorescence intensity of HA-positive cells was compared. ***P<0.001

Tethering receptors can bind to and thereby sense apoptotic cells; however, they cannot stimulate phagocytes to ingest apoptotic cells by themselves.^14, 15, 16^ It has been hypothesized that tethering receptors transduce apoptotic cell recognition signals into phagocytes via their interacting co-receptor. To test whether the co-receptors of tethering receptors are required for tethering receptor-mediated engulfment of apoptotic cells, we generated a chimeric annexin A5 (Anxa5-GPI) fused to the leader sequence of Ig κ-chain, the hemagglutinin A (HA) epitope, and the GPI anchor signal of decay-accelerating factor (DAF) for its cell surface expression and detection (Figure 1a). This chimeric Anxa5-GPI presumably does not interact with any exofacial membrane proteins involved in efferocytosis, because annexin V is normally expressed in the cytosol. Thus, it could function as a tethering receptor without a co-receptor for efferocytosis through binding to PtdSer.

After construction of the chimeric protein, we examined its expression in phagocytes. Following transfection of Anxa5-GPI, LR73 cells, which were used as phagocytes, expressed the chimeric protein, as confirmed by immunoblotting (Figure 1b). The cell surface expression of Anxa5-GPI was also validated using two different approaches. Using confocal microscopy, Anxa5-GPI stained with an anti-HA antibody was observed on the boundaries of transfected LR73 cells (Figure 1c). Similarly, when Anxa5-GPI-transfected LR73 cells were stained without permeabilization and analyzed by flow cytometry, HA-positive cells were detected (Figure 1d). These results suggest that the chimeric Anxa5-GPI protein is expressed on the outer leaflet of the plasma membrane.

Some members of the annexin family including annexin A5 are secreted into the extracellular environment via unknown mechanisms, although annexins are generally considered to be cytosolic proteins.^25, 26^ To ensure that Anxa5-GPI was expressed on the cell surface via its GPI anchor, LR73 cells expressing Anxa5-GPI were treated with phosphatidyinositol-specific phospholipase C (PI-PLC) to release GPI-anchored proteins from the plasma membrane. Anxa5-GPI expression on the surface of PI-PLC-treated cells was about 20% of that on the surface of untreated cells (Figure 1e). This suggests that Anxa5-GPI on the cell surface is expressed in a GPI anchor-dependent manner.

### Anxa5-GPI expression increases the binding of apoptotic cells

**Figure 2:**
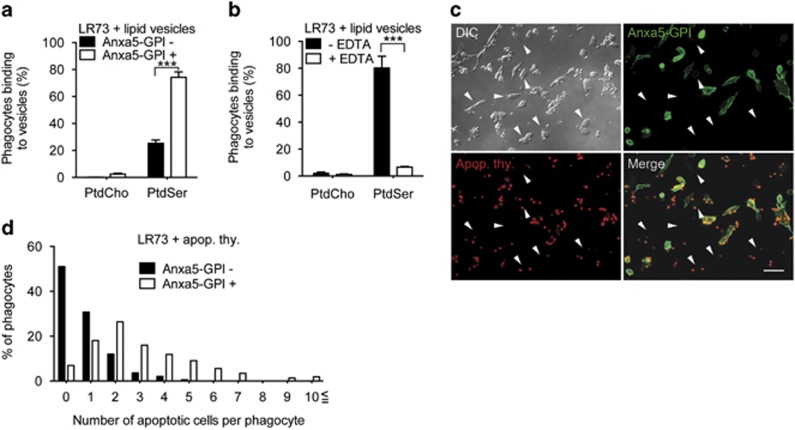
Anxa5-GPI increases binding of apoptotic cells to phagocytes. (**a** and **b**) LR73 cells transfected with Anxa5-GPI were trypsinized and incubated with NBD-PtdCho or NBD-PtdSer vesicles at 4 ^o^C for 2 h in the absence (**a**) or in the presence of EDTA (**b**). The binding of lipid vesicles to the cells was analyzed by flow cytometry. PtdCho, phosphatidylcholine; PtdSer, phosphatidylserine. (**c** and **d**) LR73 cells transfected with Anxa5-GPI were incubated with TAMRA-stained apoptotic thymocytes at 4 ^o^C for 2 h and then extensively washed with ice-cold PBS. After that, the cells were stained for Anxa5-GPI. Images were taken using fluorescence microscopy. Arrowheads indicate that cells do not express Anxa5-GPI (**c**). The number of bound apoptotic cells per phagocyte was counted from at least 100 Anxa5-GPI-positive or -negative cells from randomly selected 10 different areas (**d**). Scale bar, 40 *μ*m (**c**). ***P<0.001

Annexin A5 binds to PtdSer in a calcium-dependent manner.^26^ We next tested whether the chimera protein on the cell surface could maintain the PtdSer-binding property of annexin A5. To test this, LR73 cells transfected with Anxa5-GPI were incubated with PtdCho or PtdSer lipid vesicles at 4 °C. More than 70% of Anxa5-GPI-positive cells bound to PtdSer lipid vesicles, in comparison with 25% of Anxa5-GPI-negative cells. However, this preferential binding of Anxa5-GPI-positive cells was not observed with PtdCho lipid vesicles (Figure 2a). We next examined whether the binding of Anxa5-GPI to PtdSer lipid vesicles is dependent on calcium ions. When calcium ions were depleted from the medium using EDTA, the preferential binding of Anxa5-GPI-positive cells to PtdSer lipid vesicles was abrogated (Figure 2b). These data indicate that Anxa5-GPI on the cell surface binds to PtdSer in a calcium-dependent manner.

Next, we evaluated whether Anxa5-GPI could promote the binding of apoptotic cells to phagocytes. To test this, LR73 cells transfected with Anxa5-GPI were incubated with apoptotic thymocytes at 4 °C to inhibit the internalization, but not the binding of apoptotic cells. Many apoptotic cells were observed on Anxa5-GPI-positive cells, whereas only a few were observed on Anxa5-GPI-negative cells (Figure 2c). About 90% of Anxa5-GPI-negative cells had fewer than two tethered apoptotic cells, whereas 50% of Anxa5-GPI-positive cells had >3 tethered apoptotic cells. Surprisingly, some Anxa5-GPI-positive cells retained >10 apoptotic cells, whereas this number of apoptotic cells was never observed on Anxa5-GPI-negative cells (Figure 2d). Taken together, these data indicate that Anxa5-GPI expressed on the cell surface preserves the PtdSer-binding property of annexin A5 and enhances the binding of apoptotic cells to phagocytes.

### Anxa5-GPI-promoted binding of apoptotic cells increases efferocytosis

**Figure 3:**
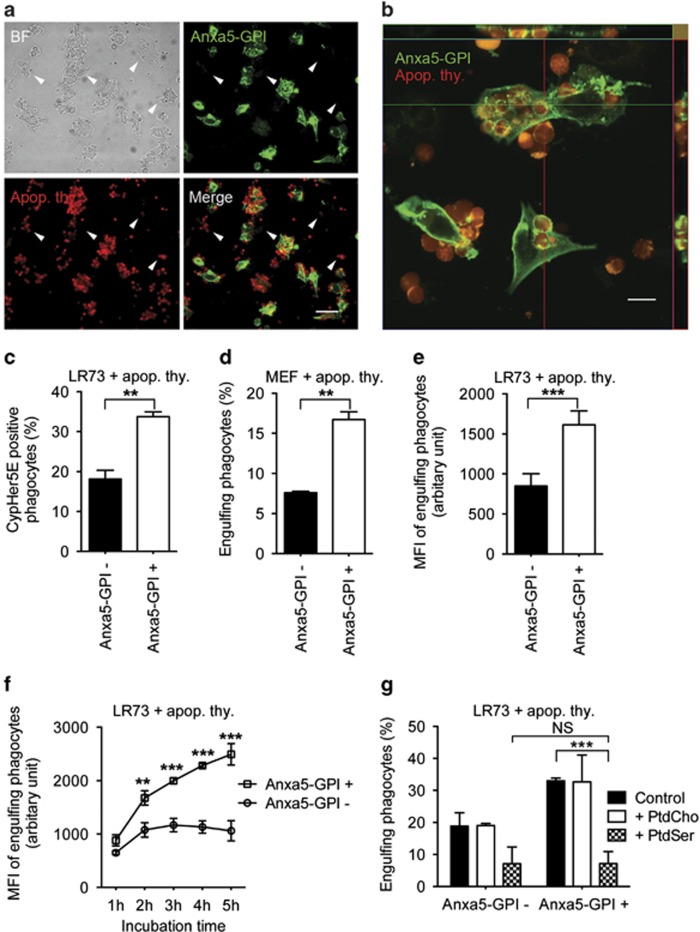
Increased binding of apoptotic cells by Anxa5-GPI enhances phagocytosis of apoptotic cells. (**a** and **b**) LR73 cells transfected with Anxa5-GPI were incubated with TAMRA-stained apoptotic thymocytes at 37 ^o^C for 2 h, extensively washed and then stained with anti-HA antibody for Anxa5-GPI. The pictures were taken using fluorescence microscopy (**a**) or confocal microscopy (**b**). Arrowheads indicated Anxa5-GPI-negative cells. Scale bar, 40 *μ*m (**a**), 10 *μ*m (**b**). (**c**) LR73 cells transfected with Anxa5-GPI were incubated with CypHer5E-stained apoptotic cells for 2 h, then stained for Anxa5-GPI and analyzed by flow cytometry. The CypHer5E and Anxa5-GPI double-positive cells were considered as Anxa5-GPI-expressed phagocytes ingesting apoptotic cells. (**d**) MEF cells nucleofected with Anxa5-GPI were incubated with TAMRA-stained apoptotic thymocytes for 2 h, stained for Anxa5-GPI and then analyzed by flow cytometry. (**e** and **f**) LR73 cells transfected with Anxa5-GPI were incubated with TAMRA-stained apoptotic cells for 2 h (**e**) or for the indicated times (**f**), washed, trypsinized, stained for Anxa5-GPI and then analyzed by flow cytometry. The TAMRA MFI of engulfing phagocytes was evaluated. (**g**) LR73 cells transfected with Anxa5-GPI were incubated with apoptotic thymocytes in the absence or presence of PtdCho or PtdSer lipid vesicles for 2 h. After that, the cells were stained for Anxa5-GPI and analyzed by flow cytometry. NS, not significant; **P<0.01, ***P<0.001

After confirming the Anxa5-GPI-enhanced binding of apoptotic cells to phagocytes, we tested whether the increased binding of apoptotic cells to phagocytes could enhance the ingestion of apoptotic cells. To test this, LR73 cells transfected with Anxa5-GPI were incubated with apoptotic thymocytes stained with TAMRA at 37 °C to allow phagocytes to ingest apoptotic cells. Intriguingly, Anxa5-GPI-positive cells contained many more apoptotic cells than Anxa5-GPI-negative cells (Figure 3a). To ensure that Anxa5-GPI-positive cells internalized apoptotic cells, confocal microscopy was performed. Most apoptotic cells on the top of Anxa5-GPI-positive cells are inside of phagocytes. Interestingly, Anxa5-GPI densely localized around apoptotic cells to form halos or phagocytic cups, which is reminiscent of other engulfment receptors (Figure 3b).^8, 11, 27, 28^ In an alternative approach, apoptotic cells stained with the pH-sensitive dye cypHer5E were used. This dye is maximally fluorescent at an acidic pH and is thus used to observe apoptotic cells in phagolysosomes. When cypHer5E-stained apoptotic thymocytes were incubated with LR73 cells transfected with Anxa5-GPI, more Anxa5-GPI-positive cells than Anxa5-GPI-negative cells were cypHer5E positive (Figure 3c). This suggests that Anxa5-GPI enhances the ingestion of apoptotic cells. This increased phagocytosis of apoptotic cells by Anxa5-GPI was not limited to LR73 cells. Anxa5-GPI also enhanced efferocytosis by mouse embryonic fibroblasts (Figure 3d), which indicates that Anxa5-GPI-promoted efferocytosis is not a cell-type-specific phenomenon.

In addition, we tested whether Anxa5-GPI could increase the rate of engulfment of apoptotic cells. Under our experimental conditions, it is possible to consider TAMRA-positive phagocytes as phagocytes internalizing apoptotic cells (Supplementary Figure 1), and the TAMRA mean florescence intensity (MFI) of engulfing phagocytes represents the relative number of apoptotic cells engulfed per phagocyte. The TAMRA MFI of Anxa5-GPI-positive phagocytes was about twofold higher than that of Anxa5-negative phagocytes, which indicates that Anxa5-GPI-positive phagocytes ingest apoptotic cells at a rate twice as great as Anxa5-GPI-negative phagocytes, and that Anxa5-GPI also promotes an increase in the number of apoptotic cells engulfed per phagocyte (Figure 3e). More intriguingly, Anxa5-GPI expression in LR73 cells continuously augmented the TAMRA MFI, whereas the MFI of Anxa5-GPI-negative phagocytes stopped increasing it after 2 h (Figure 3f), which indicates that Anxa5-GPI-positive phagocytes successively engulf apoptotic cells but not Anxa5-GPI-negative phagocytes, which is similar to other engulfment receptors.^29^ These data suggest that Anxa5-GPI enhances the rate and efficiency of efferocytosis and also enables phagocytes to uptake apoptotic cells consecutively.

To test whether Anxa5-GPI-induced efferocytosis is PtdSer dependent, phagocytes were incubated with apoptotic cells in the presence of PtdSer or PtdCho lipid vesicles. Anxa5-GPI-induced efferocytosis was completely abolished in the presence of PtdSer lipid vesicles, but was not affected by the presence of PtdCho lipid vesicles (Figure 3g). Taken together, these data suggest that the increased binding of apoptotic cells by Anxa5-GPI enhances ingestion of apoptotic cells in a PtdSer-dependent manner and that Anxa5-GPI, an artificial tethering receptor without a putative co-receptor, is sufficient to promote efferocytosis. Furthermore, these data imply that co-receptors are dispensable for tethering receptor-mediated efferocytosis.

### Target specificity of Anxa5-GPI-mediated phagocytosis

**Figure 4:**
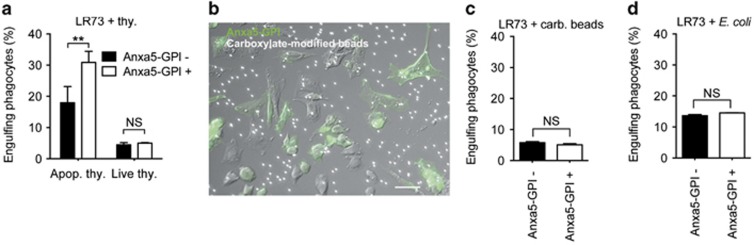
Anxa5-mediated phagocytosis is highly specific to apoptotic cells. (**a**) LR73 cells transfected with Anxa5-GPI were incubated with TAMRA-stained live or apoptotic thymocytes for 2 h, stained with anti-HA antibody, and then analyzed by flow cytometry. HA and TAMRA double-positive cells were considered as engulfing phagocytes. (**b** and **c**) Anxa5-GPI-transfected LR73 cells were incubated with 2 *μ*m red fluorescent carboxylate-modified beads for 2 h and stained for Anxa5-GPI. Engulfing phagocytes were analyzed by fluorescent microscopy (**b**) or flow cytometry (**c**). Scale bar, 20 *μ*m (**b**). (**d**) LR73 cells transfected with Anxa5-GPI were incubated with dead fluorescent *Escherichia coli* for 1 h. *E. coli* and Anxa5-GPI double-positive cells were considered as the cells expressing Anxa5-GPI and phagocytosing *E. coli*. NS, not significant; **P<0.01

Many tethering receptors and engulfment receptors have a wide range of target specificities from pathogens to apoptotic cells. Next, we investigated the specificity of Anxa5-GPI-mediated phagocytosis. First, we tested whether Anxa5-GPI specifically enhances phagocytosis of apoptotic cells, not of live cells. As expected, Anxa5-GPI did not promote the phagocytosis of live cells but strongly enhanced the engulfment of apoptotic cells (Figure 4a). We also examined whether Anxa5-GPI promoted the engulfment of carboxylate-modified beads, which are surrogate targets of apoptotic cells because they mimic the negative surface charge of these cells. Accordingly, they have been used instead of apoptotic cells to study efferocytosis.^30, 31, 32^ PtdSer-sensing receptors such as BAI1 and Tim-4 reportedly promote the phagocytosis of these beads.^8, 14^ Nonetheless, Anxa5-GPI enhanced neither binding nor ingestion of carboxylate-modified beads (Figures 4b and c). In addition, the effects of Anxa5-GPI on the phagocytosis of *E. coli* were tested. Anxa5-GPI failed to enhance the phagocytosis of *E. coli* (Figure 4d). Taken together, these data indicate that Anxa5-GPI-mediated phagocytosis is highly specific to apoptotic cells, unlike phagocytosis mediated by other PtdSer-sensing receptors.

### Anxa5-GPI-induced engulfment is partially dependent on a specific engulfment pathway

**Figure 5:**
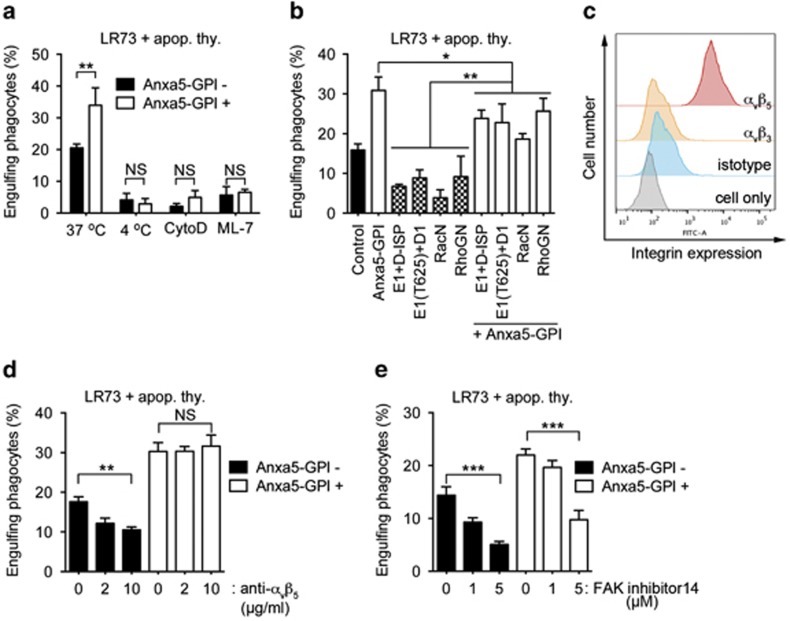
Anxa5-GPI-induced engulfment of apoptotic cells is partially dependent on a specific engulfment pathway. (**a**) Apoptotic thymocytes were incubated with LR73 cells transfected with Anxa5-GPI at 37 ^o^C, 4 ^o^C or in the presence of cytochalasin D or ML-7 for 2 h and engulfing phagocytes were analyzed by flow cytometry. CytoD, cytochalasin D. (**b**) LR73 cells were transfected with the indicated plasmids, incubated with apoptotic cells for 2 h and analyzed by flow cytometry. D, Dock1; E1, Elmo1. (**c**) LR73 cells were stained with antibodies against *α*_v_*β*_3_ or *α*_v_*β*_5_ and analyzed by flow cytometry. (**d**) LR73 cells transfected with Anxa5-GPI were pre-incubated with anti-*α*_v_*β*_5_ antibody for 20 min. After that, apoptotic thymocytes were added and incubated for 2 h. Engulfing phagocytes were measured by flow cytometry. (**e**) Anxa5-GPI-transfected cells were incubated with apoptotic thymocytes in the absence or presence of FAK inhibitor 14 for 2 h. Phagocytes ingesting apoptotic cells were evaluated using flow cytometry. NS, not significant; *P<0.05, **P<0.01, ***P<0.001

Next, we examined the mechanisms by which Anxa5-GPI enhances efferocytosis. We first tested whether Anxa5-GPI-mediated efferocytosis is dependent on the cytoskeletal engulfment machinery. To test this, LR73 cells transfected with Anxa5-GPI were incubated with apoptotic cells at 4 °C (blocking ATP-dependent processes and thus the internalization of apoptotic cells) or in the presence of cytochalasin D (inhibitor of actin polymerization) or ML-7 (inhibitor of myosin light-chain kinase). Anxa5-GPI-induced efferocytosis was abrogated at 4 °C and in the presence of these inhibitors (Figure 5a), which suggests that Anxa5-GPI-induced efferocytosis relies on the known cytoskeletal engulfment machinery.

We next tested the dependence of Anxa5-GPI-mediated efferocytosis on a specific engulfment pathway. LR73 cells co-expressing Anxa5-GPI and dominant-negative forms of Elmo1, Dock1, Rac1, and RhoG (namely, E1(T625), Dock-ISP, RacN, and RhoGN, respectively) were incubated with apoptotic cells. All dominant-negative proteins substantially inhibited the basal uptake of apoptotic cells, but none abolished Anxa5-GPI-promoted efferocytosis. Intriguingly, Anxa5-GPI-induced engulfment was most severely inhibited by RacN and was partially suppressed by the other dominant-negative proteins (Figure 5b). This might be because Rac is downstream of several signaling pathways. Thus, RacN could inhibit multiple engulfment signaling pathways.

Next, we tested what receptors function as tickling receptors for Anxa5-GPI-mediated efferocytosis. In order to address this, we first examined whether LR73 cells express several known PtdSer receptors, Tim-4, BAI1 and Stabilin-2. None of these receptors were detectable in LR73 cells (Supplementary Figures 2a and c). These data exclude that these receptors function as ticking receptors for Anxa5-GPI-mediated efferocytosis in these cells. Integrins such as *α*_v_*β*_3_ and *α*_v_*β*_5_ function as engulfment receptors for apoptotic cells. Moreover, it was recently reported that integrin *α*_v_*β*_3_ mediates Tim-4-induced efferocytosis.^30, 33, 34^ Thus, we tested the expression of the integrins. *α*_v_*β*_5_ was highly expressed in LR73 cells, whereas *α*_v_*β*_3_ was undetectable (Figure 5c).^35^ Then, we tested whether integrin *α*_v_*β*_5_ mediates Anxa5-GPI-induced engulfment and thus functions as a tickling receptor in these cells. Interestingly, a blocking antibody against *α*_v_*β*_5_ failed to inhibit Anxa5-GPI-promoted engulfment, although it inhibited the basal engulfment of apoptotic cells in a dose-dependent manner (Figure 5d). By contrast, Anxa5-GPI-promoted engulfment was abolished when focal adhesion kinase (FAK) was blocked using FAK inhibitor 14 (Figure 5e), which indicates that various integrins rather than a specific integrin is involved in Anxa5-GPI-mediated efferocytosis. Taken together, these data imply that Anxa5-GPI-induced efferocytosis is dependent on the known cytoskeletal engulfment machinery but simultaneously utilizes multiple engulfment signaling pathways.

### Production of cytokines upon Anxa5-GPI-induced engulfment of apoptotic cells

**Figure 6:**
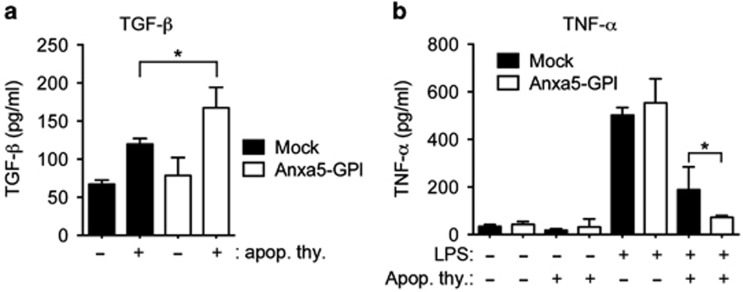
Anxa5-GPI-induced efferocytosis causes anti-inflammatory responses. (**a**) Mock- or Anxa5-GPI-transfected LR73 cells were incubated with apoptotic thymocytes in serum-free alpha-MEM for 18 h and then the conditioned medium were collected. TGF-*β* in the medium was measured using ELISA. (**b**) LR73 cells transfected with Anxa5-GPI were incubated with apoptotic thymocytes in serum-free alpha-MEM in the presence or absence of LPS or apoptotic thymocytes for 18 h. Supernatants were evaluated for TNF-*α* cytokine production by ELISA. *P<0.05

Efferocytosis is an immunologically silent process, which produces anti-inflammatory cytokines and suppresses the production of pro-inflammatory cytokines. We examined whether Anxa5-GPI-mediated efferocytosis also results in anti-inflammatory responses. Mock- or Anxa5-GPI-transfected cells produced a comparable level of TGF-*β* in the absence of apoptotic cells. However, more TGF-*β* was produced in Anxa5-GPI-transfected cells than in mock-transfected cells when apoptotic cells were added (Figure 6a). Moreover, Anxa5-GPI-mediated efferocytosis suppressed lipopolysaccharide (LPS)-induced TNF-*α* production. Both mock- and Anx5-GPI-transfected cells treated with LPS exhibited substantial TNF-*α* production; however, TNF-*α* production was inhibited by incubation with apoptotic cells. LPS-induced TNF-*α* production was more effectively suppressed in Anxa5-GPI-transfected cells than in mock-transfected cells (Figure 6b), which suggest that Anxa5-GPI-mediated efferocytosis leads to anti-inflammatory responses, similar to efferocytosis executed by other engulfment signaling pathways.

In conclusion, the data presented here suggest that co-receptors are unnecessary for tethering receptor-mediated efferocytosis, and that tethering receptors simultaneously exploit multiple engulfment signaling pathways to promote phagocytosis of apoptotic cells.

## Discussion

Phagocytes sense apoptotic cells by receptors of the former cells recognizing ligands on the latter cells. After recognizing apoptotic cells, the receptors on phagocytes directly or indirectly transduce apoptotic cell recognition signals into phagocytes to initiate the internalization of apoptotic cells. Receptors that indirectly transduce these signals are known as tethering receptors. It has been suggested that tethering receptors can mediate efferocytosis through co-receptors or other engulfment receptors located nearby, known as two-step engulfment. In two-step engulfment, tethering receptors secure apoptotic cells on phagocytes, after which other engulfment receptors bind to apoptotic cells and transduce apoptotic cell recognition signals into phagocytes to mediate the phagocytosis of the tethered apoptotic cells.^12, 33^ However, in co-receptor-mediated engulfment, it is still unclear whether the co-receptors exist or are essential for tethering receptor-mediated efferocytosis.

To address this question, we generated Anxa5-GPI to mimic a tethering receptor that does not have a putative co-receptor. Presumably, no proteins interact with Anxa5-GPI on the cell surface, because annexin A5 is normally expressed in the cytosol (Supplementary Figure 3). However, we could not completely exclude the possibility that Anxa5-GPI associates with a protein on the cell surface. Annexin A5 might be secreted, although it is mainly cytosolic, which increases the likelihood that Anxa5-GPI associates with an extracellular protein or a plasma membrane protein. However, if this does occur, the protein Anxa5-GPI associates with might not be a co-receptor for efferocytosis but for other annexin A5-mediated functions because of the inhibitory effect of annexin A5 on efferocytosis. Therefore, although Anxa5-GPI might interact with a protein in the extracellular environment, this protein should not function as a co-receptor for Anxa5-GPI-mediated efferocytosis.

Tim-4 is a well-known PtdSer-binding protein and is proposed to be a tethering receptor because its cytoplasmic tail and transmembrane domain are not necessary for efferocytosis. Recent studies of Tim-4 have focused on the mechanisms by which Tim-4 enhances the engulfment of apoptotic cells.^33, 36, 37, 38^ These studies suggested that integrins or Mertk are involved in Tim-4-mediated engulfment of apoptotic cells. However, they failed to clearly determine whether these are co-receptors of Tim-4. Our data imply that tethering receptors such as Tim-4 can promote phagocytosis of apoptotic cells without their co-receptors. Thus, integrins and Mertk might be bystanders that assist efferocytosis at later stages, but are not co-receptors. Nonetheless, efferocytosis was promoted more by Tim-4 than by Anxa5-GPI, although Anxa5-GPI exhibited stronger binding to PtdSer lipid vesicles than Tim-4 (unpublished data). This indicates that additional machinery facilitating apoptotic cell degradation or co-receptor machinery could exist.

Recently, it is reported that Stabilin-2, a PtdSer receptor, can crosstalk to *α*_v_*β*_5_ integrin although it transduces signals to Gulp through its cytoplasmic tail. Stabilin-2 interacts with the *β*_5_ subunit of the integrin and propagates signals through it, which further enhances Stabilin-2-mediated efferocytosis.^35^ Thus, integrins could be crucial proteins that not only function as engulfment receptors but also mediate efferocytosis induced by other engulfment receptors. These might explain that a FAK inhibitor severely affects Anxa5-GPI-mediated efferocytosis. Therefore, it is plausible that other integrins besides *α*_v_*β*_5_ function as tickling receptors for Anxa5-GPI-mediated engulfment of apoptotic cells in LR73 cells.

In addition, to identify the receptors that function as tickling molecules for Anxa5-GPI-mediated efferocytosis, we verified the expression of well-known PtdSer receptors (e.g., Stabilin-2, Tim-4 and BAI1) in LR73 cells. None of these receptors were detectable in the cells, which suggests that other unidentified engulfment receptors including integrins might be expressed in the cells and function as tickling receptors during Anxa5-GPI-mediated efferocytosis. Therefore, it seems that various unidentified receptors functioning as tickling receptors are involved in Anxa5-GPI-mediated efferocytosis in LR73 cells.

Multiple engulfment receptors mediate the phagocytosis of various targets such as apoptotic cells, artificial targets (e.g., carboxylate-modified beads), and pathogens. For example, BAI1 recognizes apoptotic cells, beads, and pathogens, and mediates their phagocytosis.^8, 39^ This is also the case for Tim-4. Tim-4 enhances the binding of pathogens (unpublished data), beads, and apoptotic cells, and promotes phagocytosis.^9, 14^ This could be caused by a versatile domain or multiple domains of PtdSer receptors binding to various targets. Nevertheless, unlike PtdSer receptors, Anxa5-GPI only enhances efferocytosis and fails to increase the phagocytosis of the beads and pathogens. It will be interesting to study whether a domain of a PtdSer receptor recognizes various targets.

Collectively, the findings of these studies could be used to anticipate the existence of co-receptors of tethering receptors. Furthermore, the novel artificial tethering receptor with a high specificity for PtdSer might be applied to therapeutic approaches to cure diseases caused by defects in the removal of apoptotic cells.

## Materials and Methods

### Cell cultures and transfections

293T cells were maintained in DMEM containing 10% FBS and 1% penicillin–streptomycin–glutamine and LR73 cells were cultured in alpha-MEM with 10% FBS and 1% penicillin–streptomycin–glutamine. 293T cells were transfected using the calcium phosphate method and LR73 cells were transfected with Lipofectamin 2000 (Invitrogen, Waltham, MA, USA) according to the manufacturer's protocol.

### Plasmids and antibodies

All plasmids constructed in the study were sequenced to confirm their identity. For the generation of Anxa5-GPI, a mouse annexin a5 cDNA was purchased from Openbiosystems (Huntsville, AL, USA), introduced into the pDisplay vector (Invitrogen) and fused to the last 37 amino acids of DAF (residues 311–347) for GPI anchorage of the protein. Elmo, Dock1, Tim-4, Rac1 and RhoG constructs used in this study have been previously described.^8, 14^ The antibodies used in the study were anti-FLAG (Sigma, St. Louis, MO, USA, M2), anti-GFP (Santa Cruz, Dallas, TX, USA, B-2), anti-HA (Santa Cruz, H-7), anti-*β*-actin (Cell Signaling, Danvers, MA, USA, 8H10D10), anti-*α*_v_*β*_3_ (Millipore, Billerica, MA, USA, 23C6), anti-*α*_v_*β*_5_ (Millipore, P1F6), anti-Tim-4 (Biolegend, San Diego, CA, USA, F31-5G3), anti-BAI1 (Orbigen, San Diego, CA, USA), and anti-Stabilin-2 (Abcam, Cambridge, MA, USA, ab194413). Flourochrome-conjugated anti-mouse or rabbit secondary antibodies were purchased from Life Technologies (Burlington, ON, Canada).

### Immunoblotting and immunoprecipitation

293T cells or LR73 cells were transfected with indicated plasmids and incubated for 48 or 24 h, respectively. After that the transfected cells were washed with ice-cold PBS and then lysed. Proteins in the lysates were separated by SDS-PAGE, transferred onto a nitrocellulose membrane, and detected by appropriate antibodies. For immunostaining, lysates were precipitated with the indicated antibodies, and the proteins co-immunoprecipitated were separated by SDS-PAGE and detected by immunoblotting or Coomassie blue staining.

### Immunostaining

LR73 cells were transiently transfected with the indicated plasmids on the 18-mm Ø cover glasses in a 12-well plate. One day after transfection, the cells were incubated with either 1 *μ*l of 2 *μ*m carboxylate-modified red fluorescent beads (Invitrogen) or TAMRA-stained apoptotic thymocytes in a CO_2_ incubator at 37 ^o^C for 2 h. The cells were then fixed with 4% paraformaldehyde in PBS for 15 min at room temperature (RT) and rinsed with PBS twice for 3 min. Then, the cells were blocked with blocking solution containing 3% bovine serum albumin (BSA, Bovogen, Keilor East, VIC, Australia), 0.1% Triton X-100 (Usb), 0.1% sodium azide (Sigma) and 1% HINGS (Gibco, Waltham, MA, USA) in PBS for 30 min at RT. Next, the cells were incubated with primary antibody (anti-HA, Santa Cruz, F-7) in 3% BSA in PBS at 4 ^o^C overnight. The cells then were washed with PBS for 5 min twice and incubated with secondary antibody (goat anti-mouse Alexa fluor 488, Life Technologies) for 1 h at RT. The cells were rinsed with PBS for 5 min twice and incubated with Hoechst 33342 (Invitrogen). Images were acquired using either Zeiss Axio Imager D2 or Zeiss LSM 700 (Oberkochen, Germany).

### Liposome preparation and binding assay

NBD-phospholipids in chloroform were purchased from Avanti (Alabaster, AL, USA). PtdCho or PtdCho/PtdSer mixture at a ratio of 2 : 8 was dried using Speed Vac (Hudson, NH, USA), resuspended with PBS at a concentration of 100 mM and then sonicated three times for 30 s. For the liposome binding assay, LR73 cells transfected with the indicated plasmids were trypsinized, suspended with alpha-MEM and then incubated with lipid vesicles at a concentration of 10 *μ*M in the presence or absence of EDTA at 4 °C for 2 h on a rotator. Next, the cells were washed with ice-cold PBS twice and resuspended with FACS buffer. Then, the cells were stained for Anxa5-GPI with anti-HA antibody followed by anti-mouse Alexa fluor 647 antibody (Life Technologies) and analyzed using flow cytometry. NBD and Anxa5-GPI double-positive cells were considered as phagocytes binding to lipid vesicles.

### Phagocytosis assay

LR73 cells were transiently transfected with the indicated plasmids. One day after transfection, the cells were incubated with TAMRA or CypHer5E or TAMRA and CypHer5E-stained apoptotic thymocytes, 2 *μ*m carboxylate-modified red fluorescent beads or fluorescein-conjugated *E. coli* for the indicated times. Next, the cells were extensively washed with ice-cold PBS in order to remove non-engulfed targets and trypsinized. The cells then were analyzed by flow cytometry. Double-positive cells for transfection and targets were considered as phagocyte-engulfing targets. A method for TAMRA-stained apoptotic cells has been previously described.^8^ Briefly, in order to make TAMRA-stained apoptotic thymocytes, thymocytes were acquired from 5–6-week-old C57/Bl6 mice. Thymocytes were then stained with 25 *μ*M of TAMRA-SE (Invitrogen), and apoptosis in thymocytes was induced using 50 *μ*M of dexamethasone (Calbiochem, Billerica, MA, USA) in an incubator with 5% CO_2_ at 37 ^o^C for 4 h. After that, apoptotic thymocytes were resuspended at a concentration of 5 × 10^5^ cells in 300 *μ*l of alpha-MEM and incubated with transfected cells.

### Cytokine production

LR73 cells transfected with Anxa5-GPI or control vector were incubated with apoptotic thymocytes suspended in serum-free medium for 18 h. Next, conditioned medium was collected and centrifuged. Cytokines in the medium were measured using ELISA according to manufacturer's protocol. In order to measure TNF-alpha, LR73 cells transfected with the plasmids were incubated with apoptotic thymocytes for 18 h in the presence or absence of LPS. After that, the medium was collected, centrifuged and TNF-alpha in the medium was measured using ELISA according to manufacturer's protocol.

### Statistical analysis

All data are shown as the mean±S.D. A two-tailed *t-*test was used to analyze statistical differences. Statistical significance was calculated using the GraphPad Prism 6 software (GraphPad, La Jolla, CA, USA) and significance was assumed when *P*-values were <0.05.

## Abbreviations

GPI: glycophosphatidylinositol
Anxa5: annexin A5
PtdSer: phosphatidylserine
PtdCho: phosphatidylcholine
HA: hemagglutinin A
BAI1: brain-specific angiogenesis inhibitor 1
DAF: decay-accelerating factor
PI-PLC: phosphatidyinositol-specific phospholipase C
MFI: mean florescence intensity
FAK: focal adhesion kinase
LPS: lipopolysaccharide
RT: room temperature
BSA: bovine serum albumin
