# Supplementary figures and images for: Co-receptors are dispensable for tethering receptor-mediated phagocytosis of apoptotic cells
Source: Cell Death Dis. 2015 May 28;6(5):e1772–. doi: 10.1038/cddis.2015.140 (PMC4669715; doi:10.1038/cddis.2015.140)

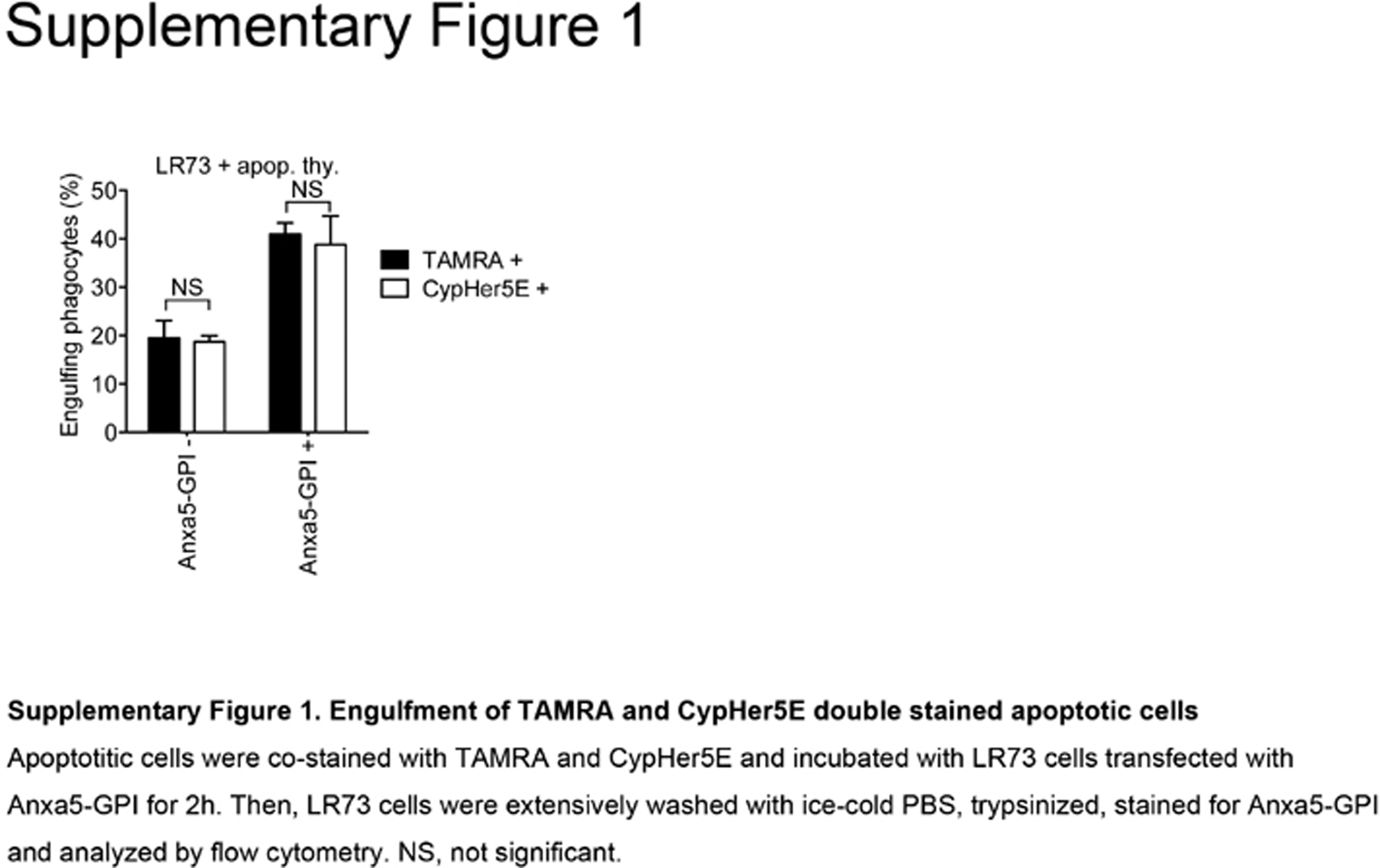

Supplement: Supplementary Figure 1 [file cddis2015140x1.tif]

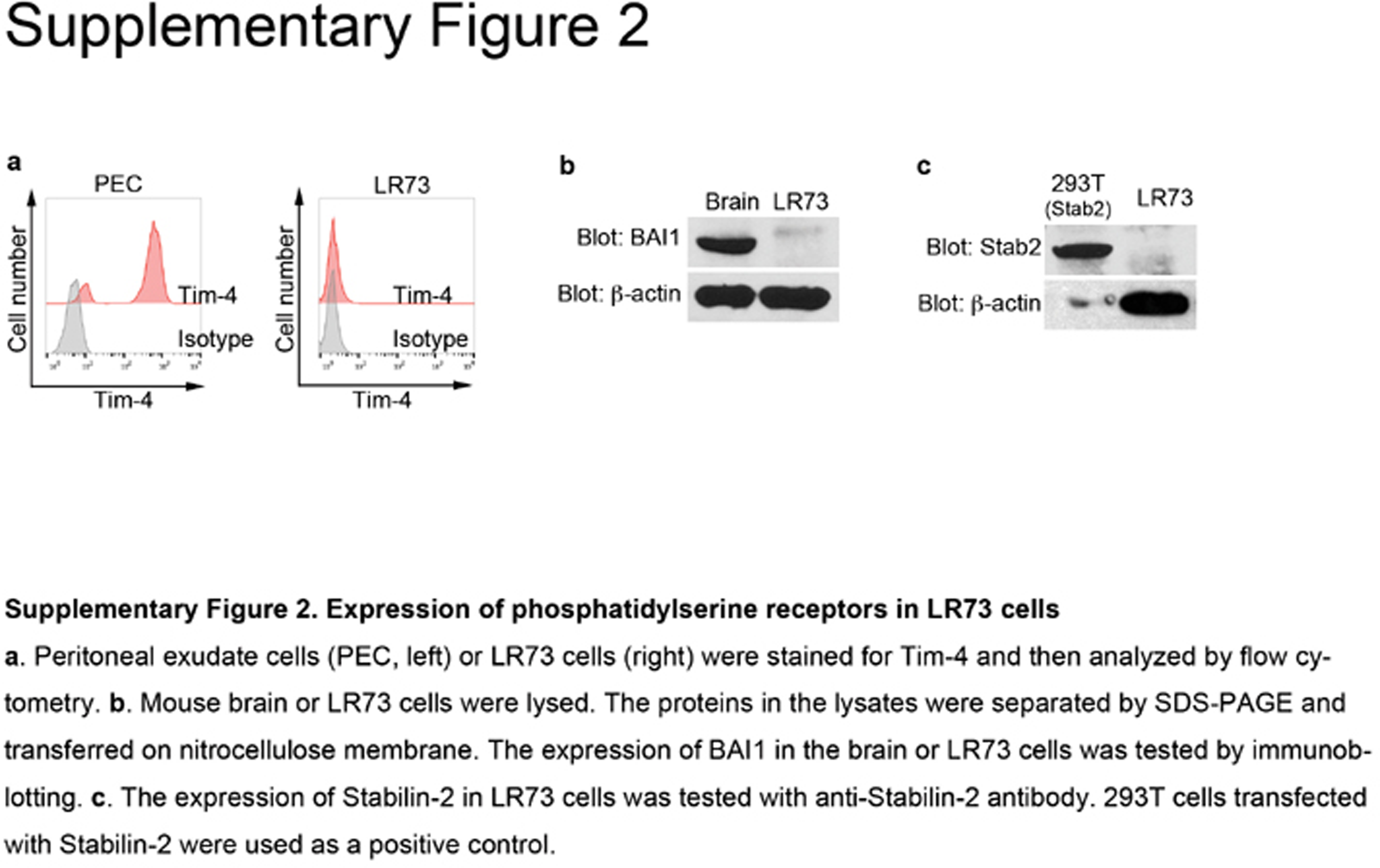

Supplement: Supplementary Figure 2 [file cddis2015140x2.tif]

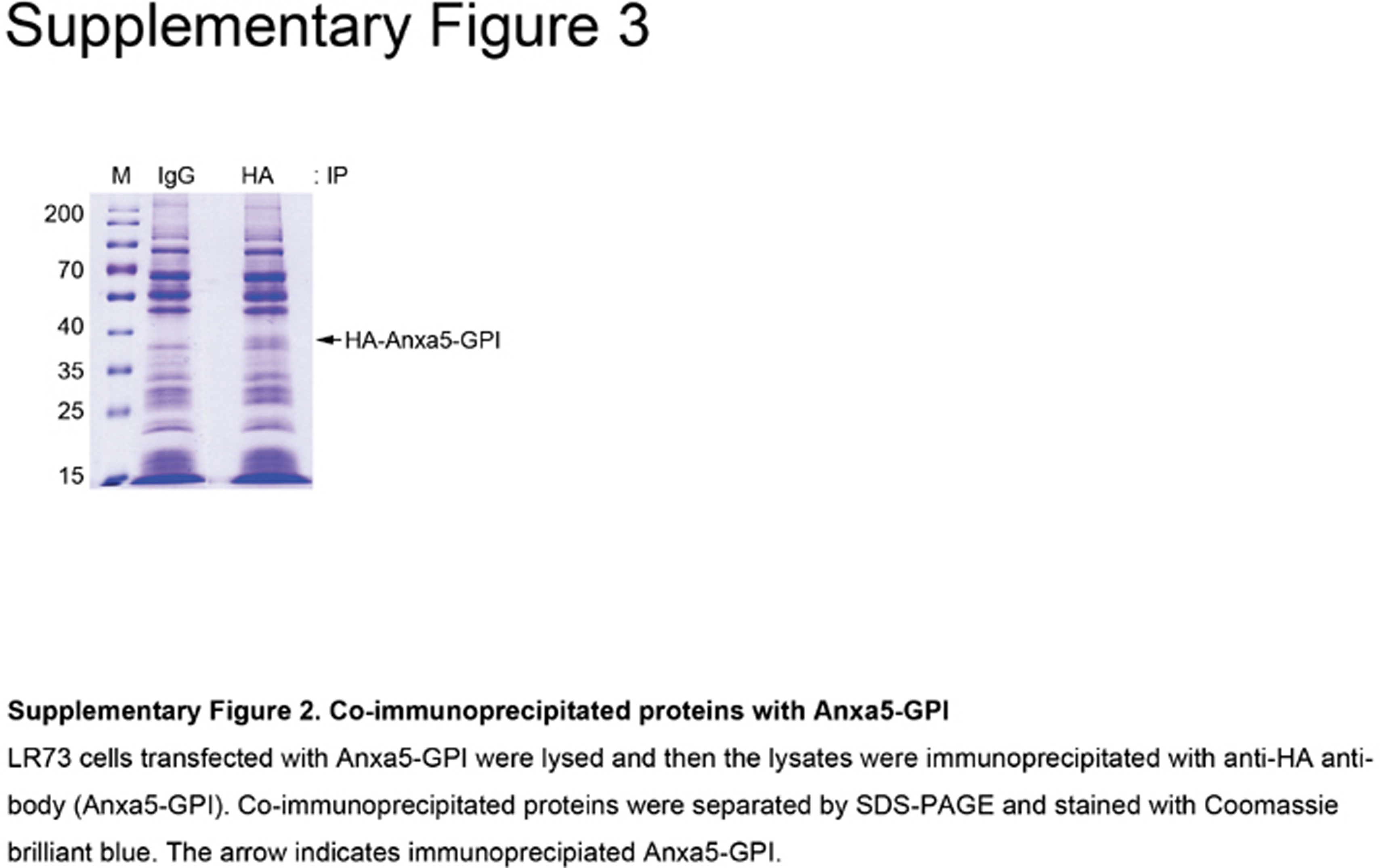

Supplement: Supplementary Figure 3 [file cddis2015140x3.tif]
